# Identification of protein profile in metacyclic and amastigote-like stages of *Leishmania tropica*: a proteomic approach

**DOI:** 10.1186/s13568-022-01481-z

**Published:** 2022-11-12

**Authors:** Marzieh Ashrafmansouri, Nasrin Amiri-Dashatan, Nayebali Ahmadi

**Affiliations:** 1 Department of Medical Laboratory Sciences, School of Paramedical Sciences, Shiraz University of Medical Sciences, Shiraz, Iran; 2 Diagnostic Laboratory Sciences and Technology Research Center, School of Paramedical Sciences, Shiraz University of Medical Sciences, Shiraz, Iran; 3 Zanjan Metabolic Diseases Research Center, Zanjan University of Medical Sciences, Zanjan, Iran; 4 Proteomics Research Center, Department of Medical Lab Sciences, Faculty of Paramedical Sciences, Shahid Beheshti University of Medical Sciences, Tehran, Iran

**Keywords:** Leishmaniasis, *Leishmania tropica*, Proteomics, SWATH-MS

## Abstract

Leishmaniasis is a tropical disease that leads to various clinical phenotypes. This study aimed to investigate protein expression changes in metacyclic and amastigote-like stages of *L. tropica* isolated from Iranian cutaneous leishmaniasis patients. Isolated samples were cultured and species type identified using PCR–RFLP technique. The promastigotes were grown in RPMI1640 media and differentiated to metacyclic and amastigote-like forms, followed by the extracted proteins of both successive stages carried out for proteomics and bioinformatics analysis. Using SWATH-MS quantitative proteomics technique, a total 176 and 155 distinct proteins were identified in metacyclic and axenic amastigote stages, respectively. Of these, 65 proteins were altered significantly (p-value < 0.05 and fold change ≥ 2) between studied stages. Several gene ontology (GO) categories were enriched for biological process during conversion of metacyclic promastigotes into amastigote-like, which “metabolic process” (GO: 0044281, P-Value: 6.52e-5), and “translation” (GO: 0006412, p-value: 5.01e–14) were disclosed as the top category in up and down-regulated proteins, respectively. Also, the KEGG pathway analysis indicated “metabolic pathways” and “ribosome” term as the most important pathways in up and down-regulated proteins, respectively. According to protein interaction network analysis, enolase (ENOL) has been detected as main hub proteins during differentiation, followed by Putative NADH-dependent fumarate reductase (LmjF.35.1180) and 40S ribosomal protein S2 (LmjF.32.0450). Overall, protein changes possibly play important roles in *L. tropica* biology. Anabolic pathways were down-regulated, whereas catabolic pathways were up-regulated during *L. tropica* differentiation. These protein expression changes could provide parasite survival in host macrophages, and could use as novel potential drug and vaccine targets for leishmaniasis.

**Graphical Abstract:**

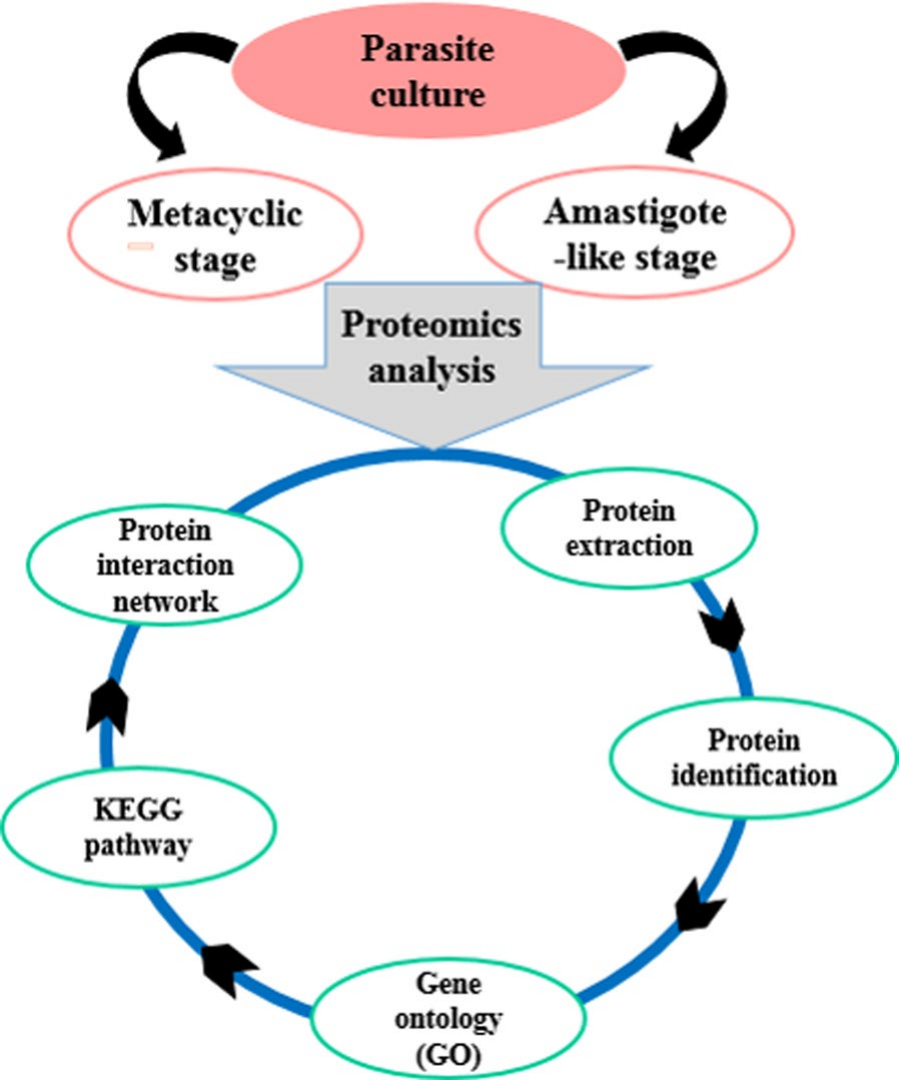

## Introduction

Parasites of the genus *leishmania* cause a wide range of disease called leishmaniasis from cutaneous lesions to fatal visceral leishmaniasis. *Leishmania* species are affecting 12 million people worldwide with 0.9–1.6 million new cases each year (Desjeux 1996, 2001). These parasites have a dimorphic life cycle including extracellular and flagellated promastigote in sndfly and an intracellular and non-motile amastigote form within the infected macrophages of vertebrate hosts. Each of promastigote and amastigote forms are adapted to residue in the different environment include midgut of the sandflies and hydrolytic environment of the phagolysosomes for a long time, respectively. Differentiation from promastigote to the amastigote accompanied by several morphological and biochemical changes which basically depends on the expression of stage-specific proteins (Bente et al. 2003). There is no vaccine for leishmaniasis and the control of these protozoa relies only on chemotherapy. The first-line of treatment relies on pentavalent antimony (SbV) compounds and drug resistant parasites has emerged worldwide (Kedzierski et al. 2009) such as Iran. *L. major* and *L. tropica* are the causative agents for cutaneous leishmaniasis in Iran and some of the neighboring countries (Ahmadi et al. 2013; Ashrafmansouri et al. 2015). Since the parasites regulate gene expression mainly at post-transcriptional stages, “Omics” approach including genomics, proteomics (Jardim et al. 2018; Sundar and Singh 2018), metabolomics (Atan et al. 2018) along with bioinformatics analysis (Dashatan et al. 2018) is thought to yield critical insight into the mechanisms of stage differentiation, parasite biology, species differences, virulence and drug resistance (Amiri-Dashatan et al. 2018, 2020a; Menezes et al. 2013; Moreira et al. 2014). In the field of the molecular differences between procyclic, metacyclic and amastigote forms, several investigation was reported the proteome of promastigotes and amastigotes forms of *L. major*, *L. infantum*, *L. donovani* and *L. Mexicana*. Most of these reports have used 2DE map to detect global differences of *Leishmania* species life stages (Amiri-Dashatan et al. 2020b; Ashrafmansouri et al. 2019), which may be due to post-translational modifications (PTMs) affect charged amino acids (Rosenzweig et al. 2008). Therefore, gel free approaches provide a valuable resource to higher proteome coverage and more precise quantitative information. Sequential window acquisition of all theoretical mass spectrometry (SWATH-MS) approach is a recently developed label free quantitative method, in which data independent acquisition is coupled with peptide spectral library match (Paape et al. 2010; Zhu et al. 2009). So far, proteomic studies to define the protein changes and pathways underlying in metacyclic and amastigote-like stages of *L. tropica* have not been reported. Identification of altered proteins during parasite development can help to introducing potential novel therapeutic targets and vaccine production for leishmaniasis. To the best of our knowledge, SWATH-based comparative proteomic analysis is the first report in Iran on the quantitative comprehensive studies regarding the proteomic profiles of metacyclic and amastigote-like of *L.tropica*. Therefore, in this study, we have employed label-free quantitative proteomics approach (SWATH) to identify differentially regulated proteins between metacyclic and amastigote-like forms in Iranian isolates of *L. tropica* by proteomic and bioinformatics approach.

## Materials and methods

### Sample collection

A total of 5 *Leishmania tropica* isolates were collected from patients in Bam city of Kerman province which is endemic region for cutaneous leishmaniasis caused by *L. tropica*. In addition, age, gender, lesion site and diameter of lesion matched participated in our study. We utilized five Iranian isolates of *L.tropica*, whom their cutaneous leishmaniasis newly diagnosed. This study was approved by Ethics Committee of Shahid Beheshti University of Medical Sciences (Ethical code: IR.SBMU.MSP.REC.1398.040). Informed consent was received from all participating patients in the present study. The identities of the isolates performed by using PCR–RFLP technique, in which the internal transcribed-spacer-1 (ITS1) region of the parasites’ ribosomal-RNA gene amplified, followed by *HaeIII* digestion of the resulting amplicons, as described previously. To carry out the PCR, we used the primers L1TSR (5ʹ-CTGGATCATTTTCCGATG-3ʹ) and L 5.8 (5ʹ-TGATACCACTTATCGCACTT-3ʹ) as the forward and reverse primers, respectively. Therefore, metacyclic and amastigote-like samples pooled separately and SWATH analysis performed in three replicates. The fold changes in current study calculated between groups.

### Cell culture and differentiation of *L. topica*

Primary isolates initially were grown on Novy-Nicolle-Mc Neal (NNN) medium and for mass culture, parasites were transferred to RPMI1640 medium (Gibco, Germany) supplemented with %10 FCS (Gibco, Germany), 100 U/ml penicillin, and 100 µg/ml streptomycin (Gibco, Germany) in 25 °C. Promastigotes were cultured with repeated medium for 6–10 days for achievement the metacyclic promastigotes form. During this time, the numbers of parasites were counted with light microscope. The parasites of stationary phase were then divided into the two aliquots. The content of one aliquot contain 10^7^ metacyclic cell/ml were centrifuged at 3500 rpm for 20 min at 4 °C and washed three times with sterile PBS (pH: 7.4) and collected in -70˚C for the protein extraction. The other aliquot was used to achieve amastigote-like parasites. To generate amastigote-like form, the cell’s environment condition was changed. Briefly, metacyclic promastigotes were placed in RPMI1640 and Schneider's Drosophila medium (pH: 3.5–4.4) supplemented with 20–25% FCS (Gibco, Germany), 200 U/ml penicillin, and 200 µg/ml streptomycin (Gibco, Germany) and maintained at 35 °C with 5% CO_2_ for 96- 120 h. The cells were monitored daily for observation of lack of flagella and spherical form of cells using Giemsa staining with an optical microscope. The 10^7^ cells/ml verified amastigote-like cells by observing cell roundness, aflagellated and immobile forms; were collected in − 70˚C for the protein extraction.

### Protein extraction and SWATH-MS analysis

The 1 × 10 ^7^ cells/ml (each of metacyclic and amastigote-like forms) were dissolved in lysis buffer (containing 8MUrea, DTT, Tris–Hcl, Glycerol, Tween–20 and 1 × protease inhibitor cocktail) and incubated for 2 h at room temperature. The cell extract was centrifuged at 15,000 g for 15 min at 4 °C to remove the cell debris. Protein concentration of supernatant was measured using Bradford assay. The soluble protein extracts were precipitated according to PhenoSwitch Bioscience laboratory protocol and stored at − 70 °C in single use aliquots. LC–MS/MS was performed at PhenoSwitch Bioscience, laboratory in Sherbrooke, Canada, using ABSciex Triple TOF 5600 instrument (ABSciex, Foster City, CA, USA) equipped with an electrospray interface with a 25 μm i.d. capillary and coupled to an EksigentμUHPLC (Eksigent, Redwood City, CA, USA). All experiments were carried out in three replicates. Proteins fold with differences in greater than 2 and *p-*value < 0.05 were detected as significant altered proteins between metacyclic promastigotes and axenic amastigotes of *L. tropica*.

### Gene ontology enrichment and pathway analysis

To better TriTryp database (The Kinetoplastid Genomics Resource) (http://tritrypdb.org/tritrypdb/) was applied for gene ontology enrichment analysis. TriTrypDB is an integrated database providing access to genome-scale datasets for kinetoplastid parasites, and supporting a variety of complex queries driven by research and development needs (Aslett et al. 2009). The differentially regulated proteins between metacyclic promastigote and axenic amastigote stages were selected for Kyoto Encyclopedia of Genes and Genomes (KEGG) pathway, and enriched based on biological process, molecular function and cellular component. Pathway analysis of differentially regulated proteins was performed using STRING database (http://string-db.org) (Mering et al. 2003).

### Protein–protein interaction (PPI) network analysis

All proteins which had significantly different expressions (up-regulated, down-regulated) in amastigote-like form compared with metacyclic stage were selected for protein–protein interaction network construction. Analyzing the network properties of protein-expression data might reveal the organizational pattern of protein expression in disease, which might in turn help us to identify new potential drug targets. Protein- protein interaction network was constructed by using STRING database, was visualized using the Cytoscape 3.6.0 software (Shannon et al. 2003). CytoHubba plugin in Cytoscape were selected for high degree (hub) proteins in obtained network. Molecular Complex Detection (MCODE) used to analyze the characteristics of the networks. The MCODE algorithm is used to find densely connected regions (modules) and then to recognize seed nodes as a complex with the highest weighted vertex in each module (Bader and Hogue 2003).

## Results

### Protein changes in metacyclic promastigotes and axenic amastigotes of *L. tropica* by SWATH-MS

**Fig. 1:**
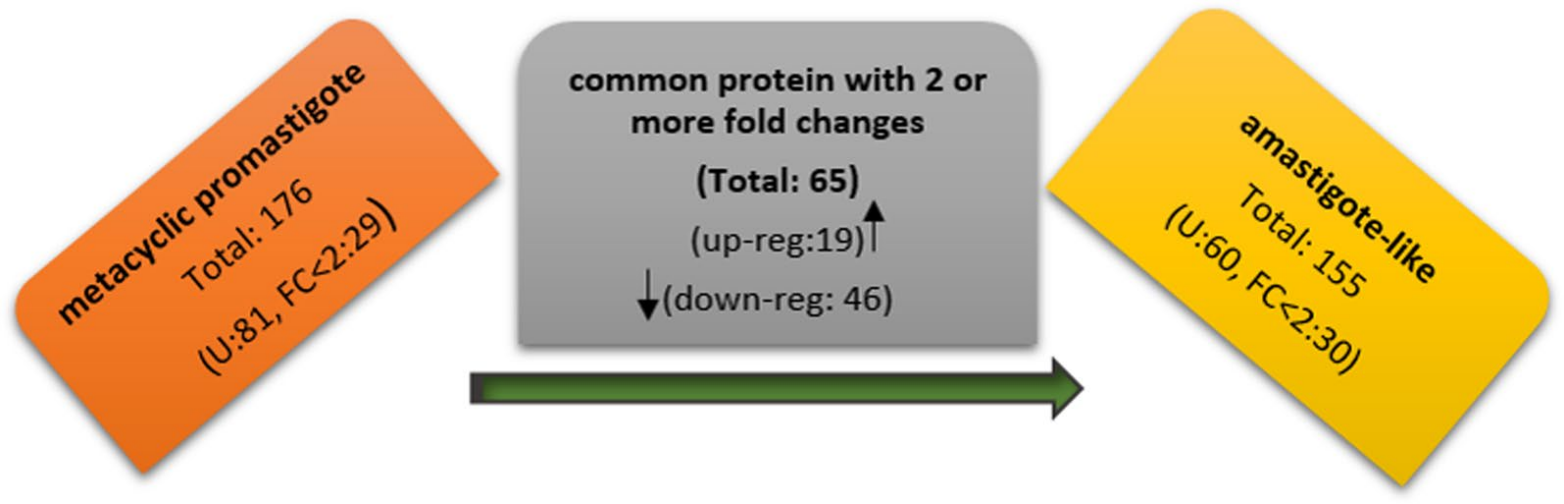
The number of protein profile, differential and uniquely protein expression between metacyclic promastigotes and axenic amastigotes of Iranian *L. tropica* isolates (U: uniquely expression; FC: Fold Change; up-reg: up-regulated; down-reg: down-regulated)

**Fig. 2:**
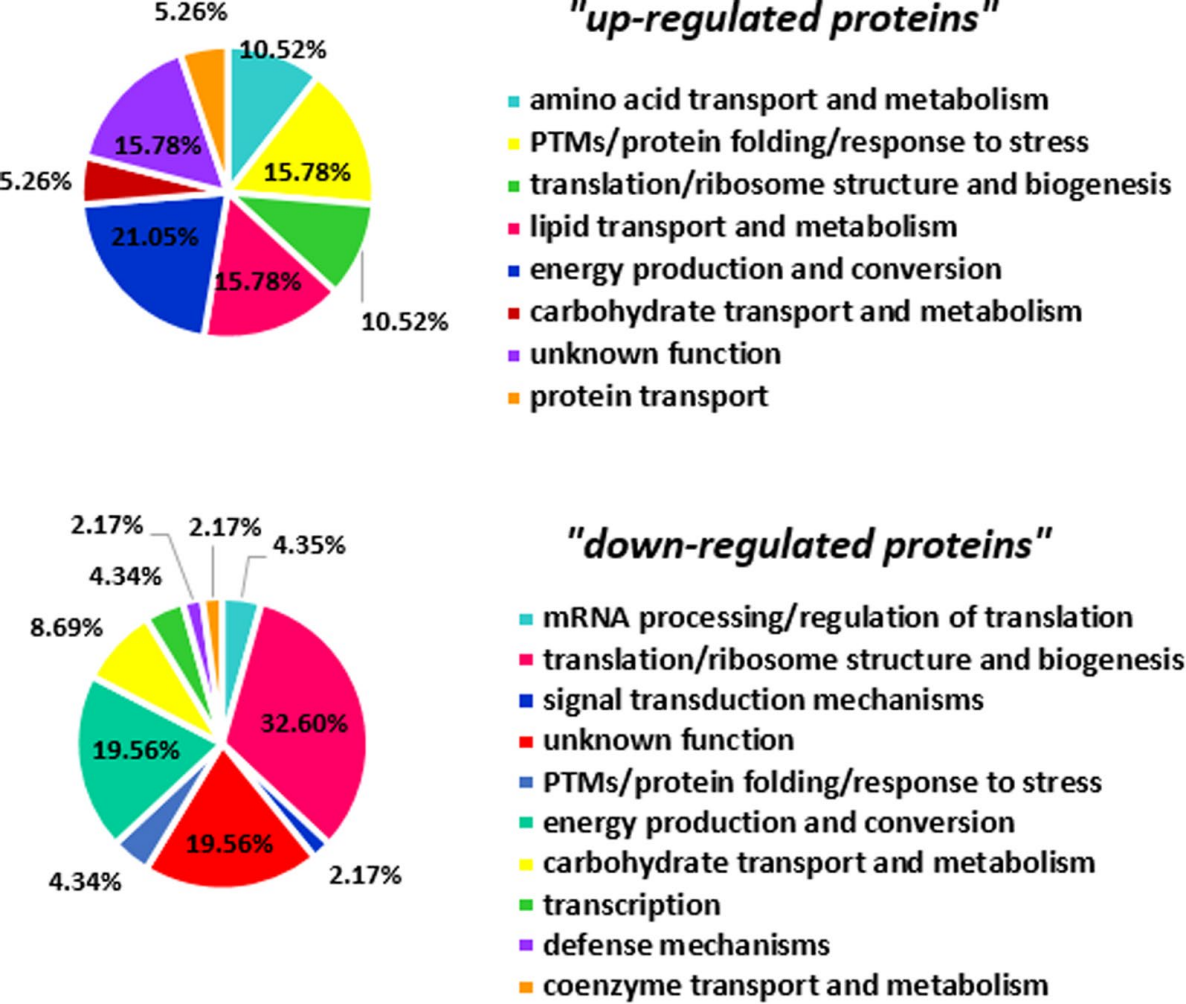
COG (clusters of orthologous groups) function classification coverage of the protein sequence. A total of 8 and 10 groups of all up-regulated & down-regulated of differentially expressed proteins were clustered by orthologous groups, respectively. PTM: post-translational modification

The significant differentially expressed proteins (fold change > 2 & p-value < 0.05) in the two developmental stages (metacyclic promastigotes and axenic amastigotes) were selected by statistical analysis. As shown in Fig. 1, a total 176 and 155 distinct proteins were identified in metacyclic and axenic amastigote stage, respectively. A total of 65 common proteins were differentially expressed in the two successive stages as up-regulated and down-regulated proteins, and detailed properties of them present in Table 1. It should be noted that 29 and 30 proteins were also expressed with fold change less than 2 in metacyclic and amastigote-like stages, respectively (Fig. 1). Among differential proteins, 19 and 46 proteins up-regulated and down-regulated during differentiation of *L. tropica* isolates, respectively (Fig. 1). One of the differential expressed proteins is hypothetical and its functions in *Leishmania* still remain to be elucidated. Further database mining indicated that the differentially expressed proteins could be classified into 18 groups based on cluster of orthologous groups of proteins (COG) function classification (Fig. 2). The COGs classification in the two developmental stages revealed that the up-regulated proteins were foremost involved in energy production and conversion cluster and down-regulated proteins were more involved in translation, ribosomal structure and biogenesis.

**Table 1:** The differential expressed proteins in *L. tropica* metacyclic and amastigote-like stages

FC AT/MT	Uniprot IDs	Protein name	Gene name	Peptides
Up-regulated proteins list during metacyclic conversion into amastigote-like form of *L. tropica*				
12.91	E9ACW0	Putative heat shock protein DNAJ	LMJF_27_2400	DIVHELPVPLEAFYCGK
7.53	E9ADS8	Putative lipophosphoglycan biosynthetic protein	LMJF_29_0760 (LPG3)	MLDILVNSLYTNR
2.05	E9AF45	Kinetoplastid membrane protein 11	LMJF_35_2210 (KMPII-1)	FAELLEQQK, LDRLDEEFNRK, EHSEHFK
3.83	Q4Q1M0	Chaperonin HSP60, mitochondrial	LMJF_36_2030	IQSIHSLLPALNHVVR, TGVTIVR,KIQSIHSLLPALNHVVR, AVAAVATTLGPK
7.28	Q4Q1R4	Putative universal minicircle sequence binding protein	LMJF_36_1610 (UMSBP1)	CGEAGHMSR
41.32	Q4Q1Y2	Putative 40S ribosomal protein S18	LMJF_36_0940	SLTLIPDHFQHIVR,FKIPDWFLNR,TEHLSSSMVDTRAGTLTAEELEKIAEIIADPAK, HAYGLR
13.02	Q4Q3V3	Succinyl-CoA:3-ketoacid-coenzyme A transferase	LMJF_33_2340	SGNLVFR, QTGGQIIR, GPGGAMDLVASGSR
21.18	Q4Q5P6	Putative 26S proteasome regulatory subunit	LMJF_32_0390	VAGLLLGR, HTNDEAIATFLAAIAR
22.5	Q4Q822	Dihydrolipoamide acetyltransferase component of pyruvate dehydrogenase complex	LMJF_28_2420	GLVVPVIR, LGLMSPFVK, NLIEDPAR
6.68	Q4Q931	Putative 40S ribosomal protein S33	LMJF_28_2420 (S33-1)	ENDMLSLMETER, GNVTQVR, LMAEAGSPDYNR
78.77	Q4Q9X6	ATP synthase subunit beta	LMJF_25_1170	IFNVLGDAIDQR, VAQSALTMAEYFR, GHGGFSVFAGVGER,FTQANSEVSALLGR, TVIIMELINNVAK
3.0	Q4QAB9	Uncharacterized protein	LMJF_24_2110	ALENPVNLDK, MEFVIDR, NEAAFQDVGIEYYR
20.39	Q4QD34	Phosphoglycerate kinase	LMJF_20_0100 (PGKC)	SALPTIQK, EGGSCILMSHLGRPK, VLGAGYAGYLMEK
2.73	Q4QDF0	Glycosomal malate dehydrogenase	LMJF_19_0710 (Gmdh)	RDPALAELAK, GSATLSMAEAGAR,VQVAGTEVVK, DPALAELAK, LLGVSLLDGLR
5.04	Q4QGX4	Putative pretranslocation protein,alpha subunit	LMJF_11_1050	QANWLMSLKPMLAVLPEIEKPR
6.15	Q4QJF1	ATPase alpha subunit	LMJF_05_0500	VDAGAPNIVSR, SPVNYNLLTGFK, FVALFNQK, VVNPLGHEVPVGL, AVDTMIPIGR
2.0	Q9U0V9	Possible 3-ketoacyl-CoA thiolase	LMJF_23_0690 (L7836.03)	LDDFTFPCLFAK, KHPDFGK
14.28	E9ACG7	Putative delta-1-pyrroline-5-carboxylate dehydrogenase	LMJF_03_0200	YGLTGAVFSR, GAFEFQGQK, CTGAVVGQQPFGGSR, GYFVEPTIIETK
9.32	E9AFE7	Putative cystathione gamma lyase	LMJF_35_3230	NNLHGGMLWFEVK, VGITDGFVR, NNLHGGMLWFEVK, NNLHGGMLWFEVK
Down-regulated proteins list during metacyclic conversion into amastigote-like form of *L. tropica*				
8.28	O62591	Probable eukaryotic initiation factor 4A	LMJF_01_0770	HNLIQGLVLSPTR, VLVTTDLVAR,HNLIQGLVLSPTR, ESLTLEGIK
28.72	Q4FX73	40S ribosomal protein S3a	LMJF_35_0400	NVLSDALVR, FTVQEVQGR, EWYDVVAPANFEK
4.81	Q4QEB3	GMP reductase	LMJF_17_0725 (GMPR)	IGVGPGSICITR, LIVGAAIGVK, GPLAPILK
2.47	Q4QG98	60S ribosomal protein L18	LMJF_13_0560 (RPL18-A)	GVDLTGISK, AAPIAVVVGDVLDDVR
2.84	E9AD27	Putative calpain-like cysteine peptidase	LMJF_27_0500	SIFLPLNTFLK, AELQRAVLKAQNAK, NATAIQDLEEALNDR
26.07	E9AD53	Putative small GTP-binding protein Rab1	LMJF_27_0760	LLLIGDSGVGK, DFADSLGIPFLETSAK
3.47	E9ADF9	Putative glycosomal phosphoenolpyruvate carboxykinase	LMJF_27_1810	VAYPLEHIPGALTHAVAGHPNNVIFLTNDAFGVMPPVAR, NLTAPELVQWALK, GALCVL SYAK, KGDVTVFFGLSGTGK, GVFNIEGGCYAK
17.39	E9ADX3	Tryparedoxin	LMJF_29_1150 (TXN2)	MPWLALPFEDRK
3.17	E9AE57	Putative fumarate hydratase	LMJF_29_1960	HGGFYLGSIGGPAAILAK, YFAHQAR, YVEEVEVFGR
2.12	E9AEB3	ATP-dependent 6-phosphofructokinase	pfk	TAIELSR, TIDNDLAFSHR, FGGTILGSSR,HLHFNPSETSIGIVTCGGICPGLNDVIR, EMVDTLVR
67.7	E9AEL4	Putative ATP-dependent DEAD-box RNA helicase	LMJF_35_0370	TASFVIPVLEK, VHILVATPGR, GFEKPSPVQEEAIPVALQGK, HIPGLEVMVTTGGTTLR, ELALQTAQVTK, NVNFEEYALR
4.36	E9AEU1	Putative NADH-dependent fumarate reductase	LMJF_35_1180	LGGNSLLECVVFGK, AATILQK, ATSGINAWGTR, LALIGGGTGVAPMLQIVR, LIGCPEANVMATLK
6.1	E9AF23	40S ribosomal protein S6	LMJF_35_2010	LFNLSR, GAIGFNTFR, RGAIGFNTFR,RVQLQDYR, VGDQPIEGVTDTTAPR
2.83	E9AFK3	Putative 60S ribosomal protein L23	LMJF_35_3790	VLNAVIIR, ISTHAPAIV, NLYVISVK
36.1	Q4Q090	2,3-bisphosphoglycerate-independent phosphoglycerate mutase	PGAM	VALQGASLVDDALK, MFVTMDR, SAEITEAAIEALK, VALQGASLVDDALK
2.64	Q4Q124	Adenosylhomocysteinase	LMJF_36_3910	AGVFFLPK, VAALHLAHVGAK, DISLAEWGR, EHVEIKPQVDR, VKDISLAEWGR, FDNLYGCR
7.9	Q4Q1D2	40S ribosomal protein S24	S24E-2	TTGFGLIYDDLASLK
2.8	Q4Q1F5	Dihydrolipoamide acetyltransferase component of pyruvate dehydrogenase complex		LTITPIPMPALSPTMEK, WFQHFHDAMENPLSLLL
2.49	Q4Q1X7	Putative 40S ribosomal protein S10	LMJF_36_0980	FFFTEGVIACK
7.71	Q4Q230	Uncharacterized protein	LMJF_36_0480	KSPIMSK, LMDQSLPVYDDVVTGVGR
2.59	Q4Q2H7	Putative vacuolar ATP synthase catalytic subunit A	LMJF_34_3670	ITWNYIR, NIVTFYEEAQR, TCLVANTSNMPVAAR, EEELQEIVQLVGK
7.61	Q4Q3U8	Putative heat shock protein	LMJF_33_2390	YNLHFNPQHPLIR, GLLPDWLR, EELTANLGTIAGSGSK
2.74	Q4Q4U1	Dihydrolipoyl dehydrogenase	GCVL-2	ALTGGVEYLFK, AAQLGLK, AVGTEDGFVK
3.42	Q4Q5P0	40S ribosomal protein S2	LMJF_32_0450	GTGIVAAPVPK, THGNLIMATFYALR
8.42	Q4Q6E1	Putative vacuolar-type proton translocating pyrophosphatase 1	LMJF_31_1220	QFQDPEVAEGR
3.81	Q4Q9H4	Putative 60S ribosomal protein L7		KILQLLR, AVEPYIAYGYPSLATVR
35.55	Q4Q9M4	Succinate–CoA ligase [ADP-forming] subunit alpha, mitochondrial	LMJF_25_2130	VIVQGMTGK, VVGGVSPK, VIVQGMTGK, AGTFHTK
3.93	Q4Q9R2	Polyprenol reductase	EnCR	DLGPQIGYR, ELESMFVHK, FSHPTMPMR
2.22	Q4Q9V1	GTP-binding nuclear protein	LMJF_25_1420	LILVGDGGTGK, SNYNFEKPFVWLAK, VCDNIPIVLVGNK
5.74	Q4Q9Y0	Putative cytochrome c oxidase VII	LMJF_25_1130	IPNPFAYSFK, VWAPATTLAEYR
5.22	Q4QAG8	Succinate dehydrogenase [ubiquinone] flavoprotein subunit, mitochondrial	LMJF_24_1630	AITMEILAGR, LGANSLLDIVVFGK, GEGGYLVNSEGER, SPVWNSNLIEALELR
18.71	Q4QAX6	Putative 60S ribosomal protein L17	LMJF_24_0040	HVQVDQAAR, SVVAMMSLLK
2.0	Q4QEI9	Elongation factor 1-alpha	LMJF_17_0080	IGGIGTVPVGR, GITIDIALWK, FESPKSVFTIIDAPGHR, SVFTIIDAPGHR, EHALLAFTLGVK, STATGHLIYK
7.48	Q4QEM2	Paraflagellar rod protein 2C	LMJF_16_1425	AQLLEHLVELVADKFR, TLGQLVYK
14.07	Q4QEX4	Putative 60S ribosomal protein L21	LMJF_16_0460	GVGVIINKPVR, TGIVWNVTPR, VGDYVDVVADSAVR
33.52	Q4QF62	60S acidic ribosomal protein P2	LMJF_15_1203	AVHIDVDQATLAFVMESVTGR, ASPSQADVEAICK
23.38	Q4QF80	Tryparedoxin peroxidase	TRYP1	GLFIIDPHGMLR
6.15	Q4QFF2	Putative ribonucleoprotein p18, mitochondrial	LMJF_15_0280	FCAMMDLMEEMQHR, FCAMMDLMEEMQHR, NCPPDLETYNATLQK
19.25	Q4QFG2	Putative 60S ribosomal protein L13a	LMJF_15_0200	APSDVFVR, HRPEIIVIDLK, HRPEIIVIDLKDHVLGR, CEQLNIAGTEIR
2.34	Q4QFL8	Enolase	ENOL	HIDEPLPILMEAIEK, LPVPCFNVINGGK
181.11	Q4QFP8	Putative small myristoylated protein-3	SMP-3	ISFEANPIAK, DNGNGLLFR
5.92	Q4QG31	40S ribosomal protein S4	RS4	LRECLPLLVIIR, AVIVTGGANR, ECLPLLVIIR,DLNNLQVTVPK, MNVIQER,DASGAEFATR
3.49	Q4QGA9	Uncharacterized protein	LMJF_13_0450	SPEFDAIYEQQQK
2.77	Q4QGC5	Tubulin alpha chain	LMJF_13_0280	LIGQVVSSLTASLR, IHFVLTSYAPVVSAEK, EIVDLALDR, QLFNPEQLVSGK, AVCMIANSTAIAEVFAR
2.59	Q4QGN9	Glucose-6-phosphate isomerase	PGI	AVLHVALR, HFVALSTNTEK, PSNSILVNALTPR, QVNLEETIFIIASK
22.93	Q4QIP1	Putative 60S ribosomal protein L7a	LMJF_07_0500	APLAVVTGLQEVTR, WPTFVTMQR,TATCVALTDVNAEDEATLK

The list of differential expression proteins based on fold change > 2 and p-value < 0.05 during developmentally process from metacyclic promastigotes to amastigotes like in Iranian L. tropica isolates

*FC* fold change, *AT* amastigote of *L. tropica*, *MT* metacyclic of *L. tropica*

### Gene ontology findings

**Fig. 3:**
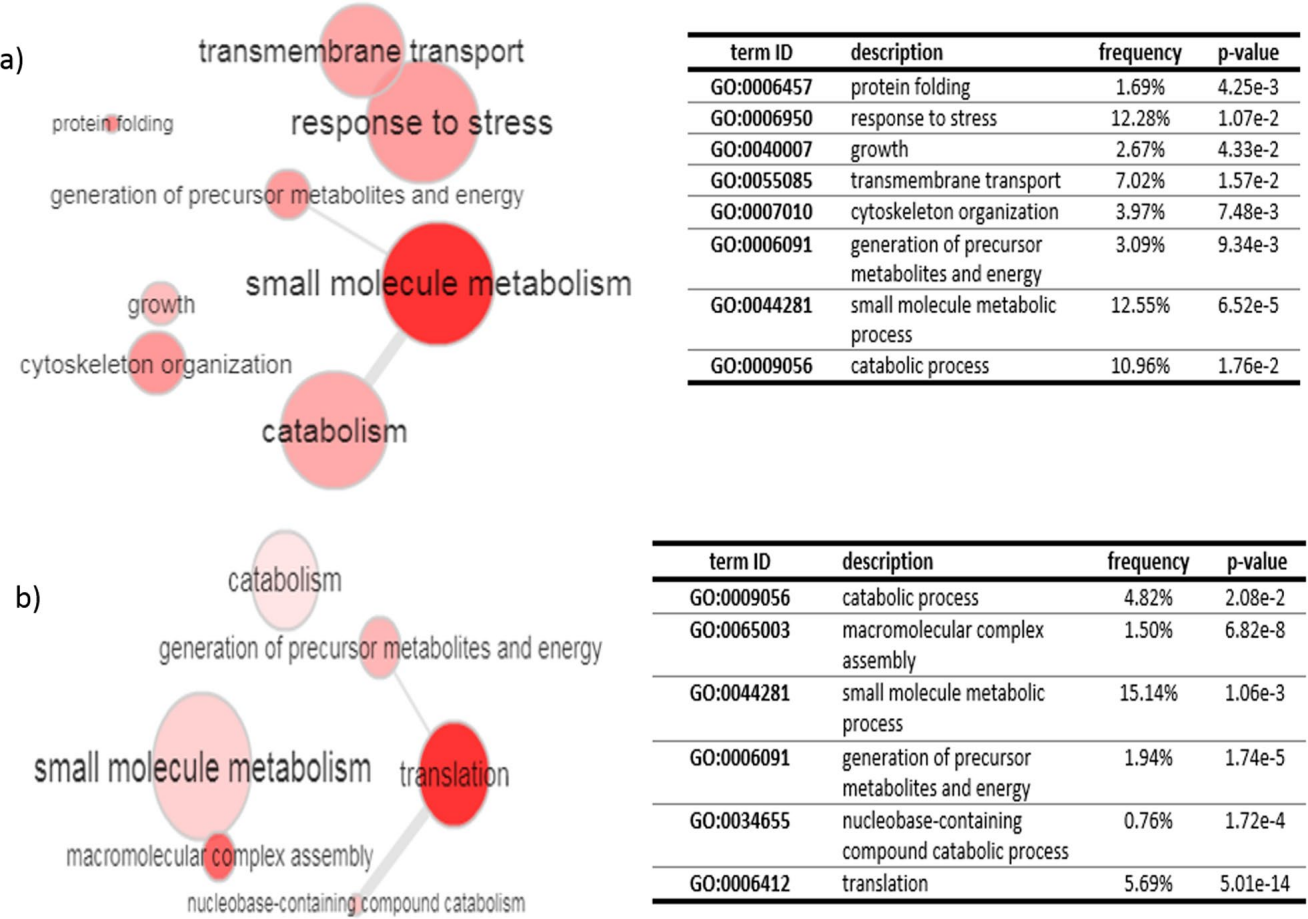
Gene ontology enrichment analysis of differentially expressed proteins based on biological process enrichment during conversion of metacyclic promastigotes into the amastigote like of Iranian isolates of *L. tropica* by TriTrypDB (kinetoplastid Genomics Resource): **a** up-regulated proteins and (**b**) down-regulated proteins. The circles color and size are corresponded on the p-value and frequency, respectively. The dark circles are related to high significance (low p-value) and large sizes are also related to higher frequency

Gene ontology (GO) analysis of total 65 significant differentially expressed proteins (up/down regulated proteins) was performed based on biological process by the kinetoplastid genomics resource database (TriTrypDB). According to gene ontology analysis of up-regulated proteins, the metabolic process (GO: 0044281), response to stress (GO: 0006950) and catabolic process (GO: 0009056) with 12.55%, 12.28% and 10.96%, had the highest frequency among other biological processes, respectively (Fig. 3a). Most of the down-regulated proteins were involved in metabolic process (1.06e-3) and translation (5.01e–14) (Fig. 3b). Total 81 and 60 proteins were uniquely expressed in metacyclic and axenic amastigote stage, respectively. Gene ontology enrichment analysis of metacyclic-specific proteins indicating that translation and response to stress had the high frequency in biological process enrichment. In addition, translation was the significant GO term in biological process of amastigote-specific expressed proteins.

### Pathway analysis

Protein expression changes (up/down regulated proteins) were also selected for KEGG pathway analysis. The pathway enrichment analysis was performed using the STRING online database. The pathway enrichment analysis revealed that the most critical pathway of up- and down-regulated proteins involved in *L. tropica* metacyclic into the amastigote-like differentiation included metabolic pathways and ribosome, carbon metabolism and glycolysis/gluconeogenesis, respectively (Table 2, 3).

**Table 2:** The KEGG pathways of up-regulated proteins during conversion of *L. tropica* metacyclic into the amastigote-like

Pathway ID	pathway description	Gene No.	FDR	Matching proteins (IDs)
1100	Metabolic pathways	7	0.0017	LmjF.03.0200,LmjF.05.0510,LmjF.19.0710,LmjF.20.0100,LmjF.23.0690, LmjF.25.1170,LmjF.28.2420
1110	Biosynthesis of secondary metabolites	4	0.0211	LmjF.19.0710,LmjF.20.0100,LmjF.23.0690,LmjF.28.2420
1200	Carbon metabolism	3	0.0251	LmjF.19.0710,LmjF.20.0100,LmjF.28.2420
20	Citrate cycle (TCA cycle)	2	0.0364	LmjF.19.0710,LmjF.28.2420
280	Valine, leucine and isoleucine degradation	2	0.0364	LmjF.23.0690,LmjF.33.2340

*FDR* false discovery rate, *KEGG* kyoto encyclopedia of genes and genomes

**Table 3:** The KEGG pathways of down-regulated proteins during conversion of *L. tropica* metacyclic into the amastigote-like

Pathway ID	Pathway description	Gene NO	FDR	Matching proteins (IDs)
3010	Ribosome	11	4.13E–10	LmjF.07.0510, LmjF.13.1230, LmjF.15.0200, LmjF.15.1207, LmjF.16.0460, LmjF.24.0040, LmjF.32.0450,LmjF.35.0420,LmjF.35.3800,LmjF.36.0990,LmjF.36.2870
1200	Carbon metabolism	9	1.76E–08	LmjF.12.0530, LmjF.14.1160, LmjF.24.1630, LmjF.27.1810, LmjF.29.1960, LmjF.29.2510, LmjF.32.3310,LmjF.36.2660,LmjF.36.6650
10	Glycolysis/gluconeogenesis	7	2.19E–08	LmjF.12.0530, LmjF.14.1160, LmjF.27.1810, LmjF.29.2510, LmjF.32.3310, LmjF.36.2660, LmjF.36.6650
1110	Biosynthesis of secondary metabolites	11	2.19E–08	LmjF.12.0530, LmjF.14.1160, LmjF.17.0725, LmjF.24.1630, LmjF.25.1770, LmjF.27.1810, LmjF.29.1960,LmjF.29.2510,LmjF.32.3310,LmjF.36.2660,LmjF.36.6650
1100	Metabolic pathways	14	2.51E–07	LmjF.12.0530, LmjF.14.1160, LmjF.15.1040, LmjF.17.0725, LmjF.24.1630, LmjF.25.1770, LmjF.27.1810, LmjF.29.1960, LmjF.29.2510, LmjF.32.3310, LmjF.34.3670, LmjF.36.2660, LmjF.36.3910,LmjF.36.6650
20	Citrate cycle (TCA cycle)	5	6.25E–06	LmjF.24.1630,LmjF.27.1810,LmjF.29.1960,LmjF.32.3310,LmjF.36.2660
620	Pyruvate metabolism	4	0.000217	LmjF.27.1810,LmjF.29.1960,LmjF.32.3310,LmjF.36.2660
190	Oxidative phosphorylation	3	0.0185	LmjF.24.1630,LmjF.31.1220,LmjF.34.3670
1230	Biosynthesis of amino acids	3	0.037	LmjF.14.1160,LmjF.29.2510,LmjF.36.6650
30	Pentose phosphate pathway	2	0.0395	LmjF.12.0530,LmjF.29.2510
260	Glycine, serine and threonine metabolism	2	0.0395	LmjF.32.3310,LmjF.36.6650

*FDR* false discovery rate

### Protein–protein interaction network analysis

**Fig. 4:**
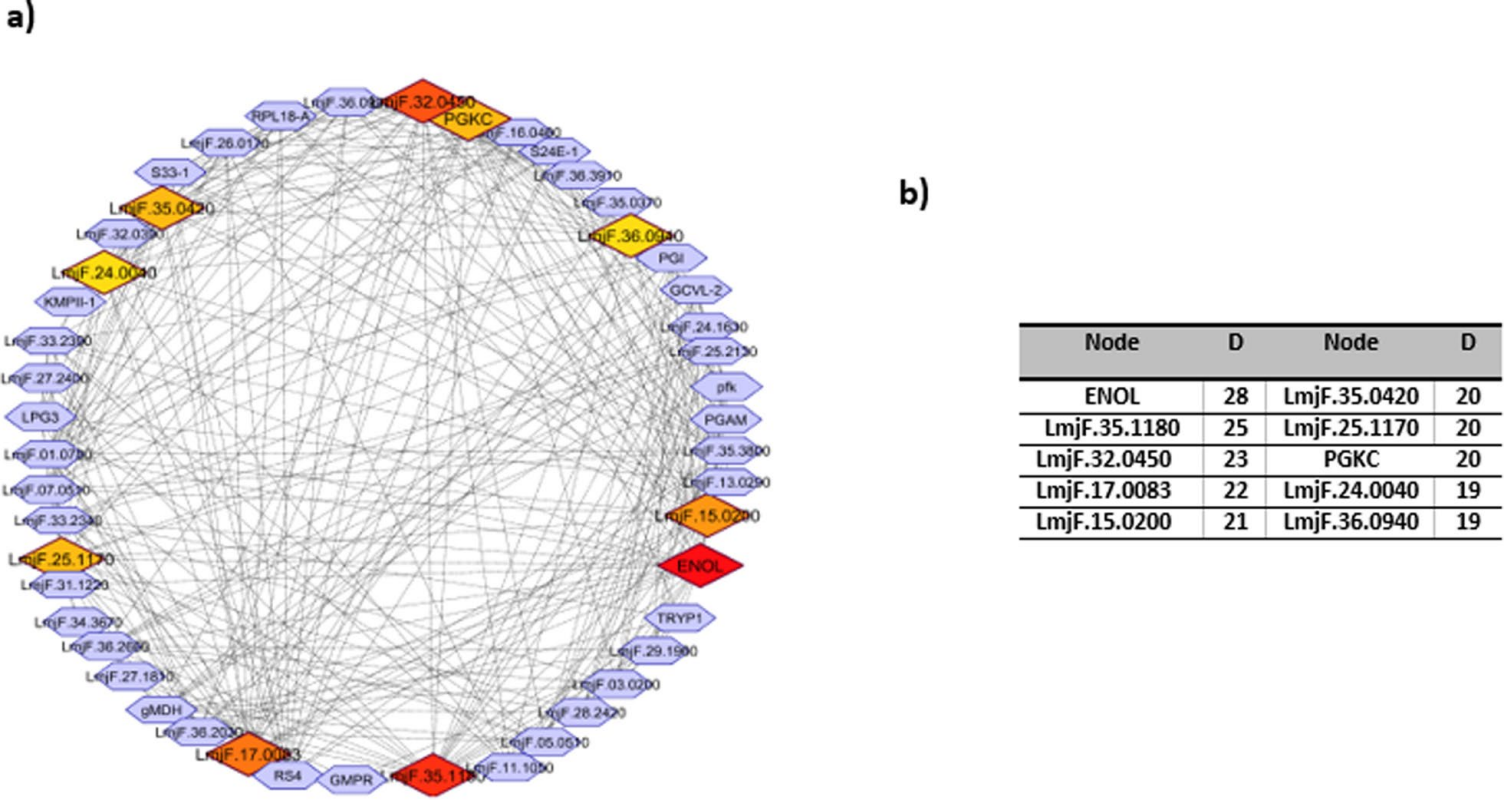
Whole Connected Component of the PPI Network of differential expressed proteins that were visualized by Cytoscape software. **a** The nodes are layout by degree value (darker to bright brown nodes corresponded to hub proteins). **b** The hub proteins along with their degree (**D**)

**Fig. 5:**
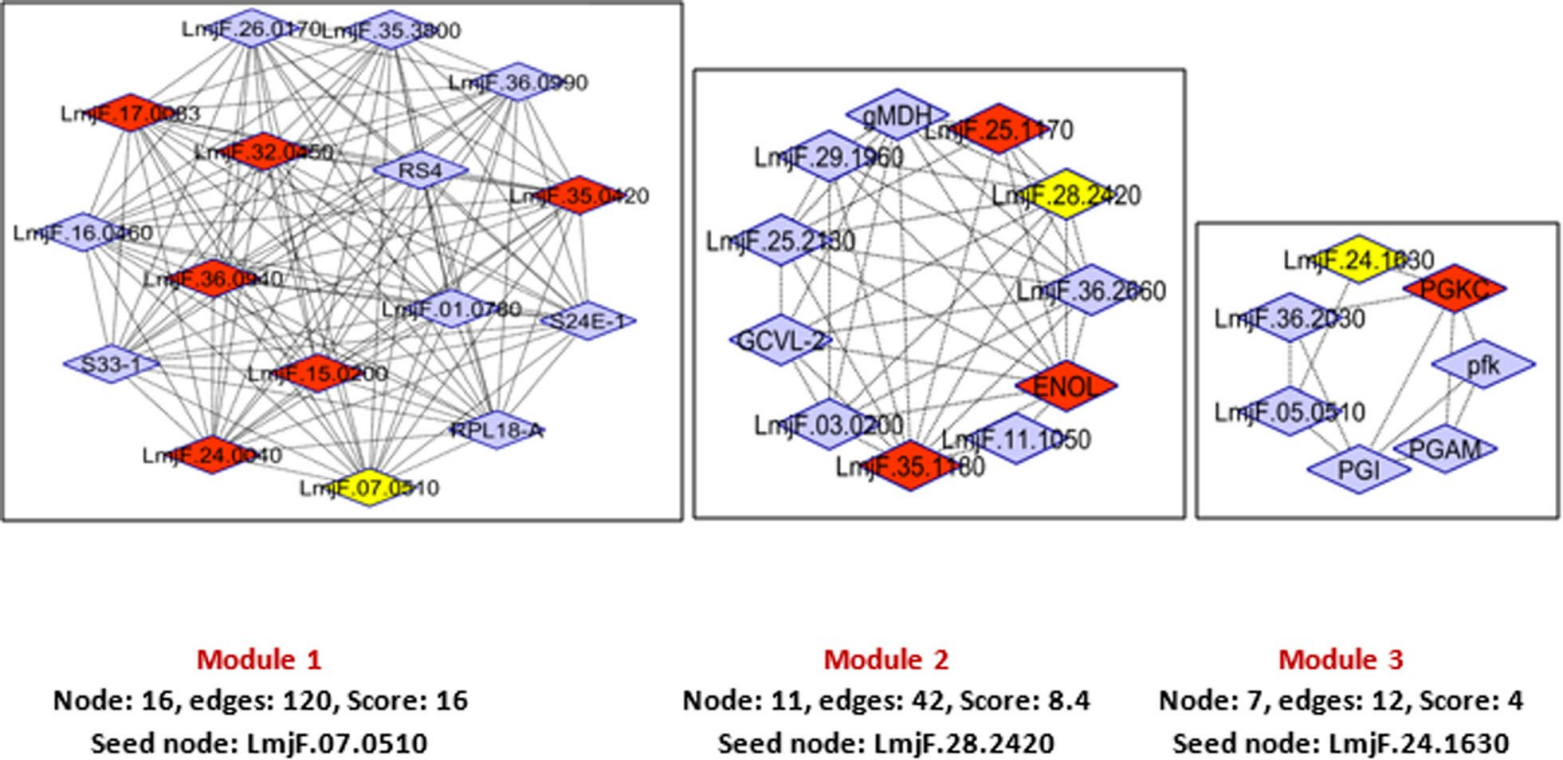
The modules of whole PPI network. The hub proteins of our analyses are presented in modules 1, 2 and 3 that are shown in orange color. Seed nodes in each module are also shown by yellow color

The PPI network of the significant differentially expressed proteins (between metacyclic and amastigote like stages of *L. tropica*) was constructed, in which including 53 nodes and 323 edges (Fig. 4a). Nodes represent the proteins from our list and others that directly interact with them. Connections contain direct interaction partners and interconnections. In order to simplify the connection patterns, interactions for the nodes with the greatest degrees (hubs) was selected. Centrality analysis based on node degree by CytoHubba (as cytoscape plugin) revealed the top 10 great number of close interconnections that can be seen with darker/different color (Fig. 4a). The hub nodes were included ENOL, LmjF.35.1180, LmjF.32.0450, LmjF.17.0083, LmjF.15.0200, LmjF.35.0420, LmjF.25.1170, PGKC, LmjF.24.0040 and LmjF.36.0940 (Fig. 4b). Further analysis of complex region of network by MCODE revealed 3 modules for the network. The seed nodes (yellow nodes in each module) of each module were included LmjF.07.0510, LmjF.28.2420 and LmjF.24.1630. The orange nodes (6, 3 and 1 node numbers in modules 1, 2 and 3, respectively) are the hub proteins that present in modules (Fig. 5).

## Discussion

In this study, we aimed to identify differentially expressed proteins between metacyclic and amastigote-like stages of Iranian isolates of *L. tropica*. To this end, we applied a quantitative proteomic approach for the first time to profile protein expression in metacyclic and amastigote-like form of *L. tropica*. To date, several transcriptomic and proteomic analyses have been reported about *Leishmania* stages. Rosenzweig et al. (Rosenzweig et al. 2008), (Lahav et al. 2011), and Saxena et al. (Saxena et al. 2007) are examples of these investigations that have studied of *Leishmania* promastigote to amastigote differentiation. A total of 176 and 155 proteins were detected in metacyclic and amastigote-like forms, respectively. Among these, 65 proteins were significantly differentially expressed between studied stages that 46 and 19 proteins were down/up-regulated in amastigote-like form, respectively. According to GO classification, the DEPs were included in various pathways (Fig. 2) that offers their vital roles in the metabolism, infectivity, virulence and pathogenicity of parasite.

Among the down-regulated proteins in our study, E9AD27, has been identified as a common protein between *L. major*, *L. tropica* and *L. infantum* isolates in Iranian patients (Hajjaran et al. 2015). At present study, another protein (Q4QFL8) has also decreased in amastigote-like form *vs* metacyclic form of *L. tropica*. This protein also reported as a differentially expressed protein between meglumine antimoniate sensitive and resistant in promastigote of *L. tropica* isolated from Iranian anthroponotic cutaneous leishmaniasis patients (Hajjaran et al. 2012). Energy production and conversion function, protein folding/response to stress and lipid metabolism were the highest rank among the up-regulated proteins in amastigote-like stage in our results. The energy production and conversion cluster were included dihydrolipoamide acetyltransferase component of pyruvate dehydrogenase complex, ATP synthase subunit beta, glycosomal malate dehydrogenase and ATPase alpha subunit proteins. Malate dehydrogenase enzyme activity links amino acid metabolism with carbohydrate metabolism pathway that led to energy production (Martin et al. 1976). According to the previous investigations, catabolic pathways that led to provide energy were also up-regulated during the *Leishmania* differentiation. Specifically, tri-carboxylic acid (TCA) cycle and respiratory chain activity were reported with highly expression in amastigote-like stage (Rosenzweig et al. 2008). Malate dehydrogenase is another up-regulated protein relate to energy production that required for performing the gluconeogenesis process in amastigote forms that is essential for amastigote proliferation within host macrophages (Naderer et al. 2006). In the present study, ATP synthase subunit beta protein up-regulated in amastigote-like and this may be essential for parasite differentiation. We found that lipid transport and metabolism involved proteins including succinyl-CoA: 3-ketoacid-coenzyme A transferase, conserved hypothetical protein and possible 3-ketoacyl-CoA thiolase also up-regulated in amastigote-like form. Succinyl-CoA: 3-ketoacid-coenzyme A transferase is a Key enzyme for ketone body catabolism that amastigote form uses these sources for energy production in absence of glucose in macrophage environment. In general, amastigote forms provide their required energy through fatty acid oxidation by elevated TCA enzymes activity and differentiating parasites shift from glucose to fatty and amino acid oxidation and from glycolysis to gluconeogenesis (Atan et al. 2018; Hart and Coombs 1982; Paape et al. 2010). In our study, the proteins involved in protein folding and response to stress increased, which is consistent with the results of previous studies. Nugent et al. reported HSP60 and HSP70 proteins in study of *L. Mexicana* differentiation (Nugent et al. 2004). Recent proteomic studies have also reported that proteins involved in stress response differentially expressed between promastigotes and amasigotes stages of L. *donovani* (Bente et al. 2003) and *L. infantum* (El Fakhry et al. 2002)*.* In addition, the up-regulated response to stress activity possibly means that amastigote form struggles with the oxidative stress to survive inside the host cells. We found that the kinetoplastid membrane protein (KMP)-11 was up-regulated during metacyclic differentiation into amastigote form. KMP-11 as a hydrophobic protein, is involved in the interaction of pathogen-host, which its expression has been reported into be increased is increased in amastigote stage (Jardim et al. 1995). According to Mukhopadhyay et al. results, the expression of KMP-11 was decreased along with parasite virulence as a function of the time of the subculture in *L. donovani* (Mukhopadhyay et al. 1998)*.* It was also reported in several independent experiments that the isolation of a Sb (III) resistant *L. infantum* cell line always correlated with a high decrease in the KMP-11 protein (El Fadili et al. 2009). In this study, based on gene ontology analysis, translation/ribosome structure and biogenesis category was the most significant cluster among the down-regulated proteins, which included several ribosomal proteins In summary, our results were in agreement with other *in-vivo* studies indicate that abundance of translation machinery proteins, translational activity and protein synthesis decreased in parasites undergoes differentiation from promastigote to amastigote (Lahav et al. 2011; Mazareb et al. 1999; Mottram and Coombs 1985). Decreased expression of mRNA processing/ replication related proteins seems during metacyclic into amastigote differentiation present beneficial since amastigotes growth and energy consumption are also slower rather than promastigotes (Mukkada et al. 1985). In addition, the down-regulation of anabolic processes involved proteins such as translational activity and glycolytic pathways and the up-regulation of catabolic functions including lipid and amino acid metabolism in amastigote-like stage were in keeping with the previous studies. Tubulin alpha chain was described as one of the down-regulated proteins in this study. This protein is a fundamental component of the cytoskeleton which is responsible for cell shape and is involved in cell division, ciliary and flagellar motility and intracellular transport. The down-regulation of this proteins indicated that the cytoskeleton organization and motility repressed in amastigote stage inside macrophage cells in mammalian host. Some uncharacterized proteins also were detected as differentially expressed proteins between studied stages that further studies are required to identify function and involved biological processes by them. In order to confirm some of the proteins identified in this study by techniques such as western blotting, we encountered limitations in the supply of the desired antibodies that were not performed. Furthermore, further in vivo and in vitro investigations are needed to identify more accurate roles of each detected proteins in differentiation, infectivity and virulence of *Leishmania*. Herein, we also investigated PPI network of differentially expressed proteins via bioinformatics approach. Since PPI network analysis is a powerful approach in categorization and ranking of the drug target candidate and potential biomarker for a certain disease (Chávez-Fumagalli et al. 2018; Dashatan et al. 2018; Flórez et al. 2010), here the PPI network of the significant different regulated proteins are constructed (Fig. 4). Topological analysis of the networks leads to rank of the nodes based on their centrality properties in network (Dashatan et al. 2018; Jeong et al. 2001). By degree centrality value using Cytohubba plugin in Cytoscape software, the top 10 node selected as important hub proteins. The hub proteins can be recommended for new potential drug targets in disease. According to results, ENOL has highest degree and this protein can be thought of as a potential drug target. Enolase described as an important enzyme in glycolysis and gluconeogenesis as two important cellular pathways. Glycolysis play important roles in ATP supply and gluconeogenesis is crucial for the virulence and viability of *Leishmania* parasite. ENOL protein plays also an important role in cell morphology and vesicle trafficking by cytoskeleton system. Furthermore, enolase enzyme is available in secretome and leishmanial parasite surface. Based on the surface enolase, plasminogen receptor can probably play a role in virulence and invasiveness of parasites (Avilán et al. 2011; Dashatan et al. 2018). It must be pointed out that further investigations are required using western blotting or real time PCR to validate the results of this study. In the present study, another hub protein with a role in energy metabolism is phosphoglycerate kinase (PGKC) and LmjF.25.1170 (ATP synthase subunit beta). Among other hub proteins, LmjF.32.0450, LmjF.17.0083, LmjF.15.0200, LmjF.35.0420, LmjF.24.0040 and LmjF.36.0940 involved in translation and are as constituents of ribosome. Therefore, manipulation and controlling of translation process in *L. tropica* could be as an approach in differentiation of parasite and also as a potential drug target to cutaneous leishmaniasis therapy. The other detected hub protein was LmjF.35.1180, NADH-fumarate reductase. NADH-fumarate reductase enzyme is an important component in the intermediate metabolism in the *Leishmania* parasite and absent in mammalian cells, furthermore, it could be a potential drug target for leishmaniasis. Module is a part of a network with closely part of proteins, which having specific biological function (Newman 2006). In this study, we demonstrated three modules in PPI network. Functional enrichment analysis of these modules showed that ATP synthesis, glycolysis/gluconeogenesis, biosynthesis of amino acids, pentose phosphate pathway, TCA cycle, translation and gene expression are the main affected pathways by differentially expressed proteins. We also categorized the modules based on the presence of hub proteins in them to get a better molecular view of parasite differentiation. The module number 1 contained the largest number of hub proteins that recognized as a hub module. The hub module proteins play possibly a more important role in parasite biology including metacyclic into amastigote differentiation. The proteins of module 1 involved in the translation and gene expression pathway, therefore, it can be concluded that the protein synthesis process is the most important pathway altered during parasite differentiation. In the current study, further analysis of modules by MCODE revealed seed nodes in modules that included LmjF.07.0510, LmjF.28.2420 and LmjF.24.1630. These seed nodes can serve as candidate drug and vaccine for cutaneous leishmaniasis caused by *L. tropica*.

In conclusions, this study presents an initial attempt at making comparisons between the global protein expression patterns of two distinct life stages (metacyclic and amastigote) of *L. tropica* species in Iranian isolates. There are very limited data on protein profile of *L. tropica*, furthermore, we showed that protein expression profiles modulated different in two successive developmentally forms of *L. tropica* using a quantitative proteomics approach (SWATH-MS). Also, several important proteins signatures introduced in sand-fly and mammalian host of *L. tropica* such as parasite biology, infectivity and pathogenesis factors, and survival in macrophage cells, which would be useful to identify potential drug targets. However, many investigations are needed to better understand the role of each differential expressed proteins to clarify molecular mechanisms of parasite differentiation. Finally, quantitative proteomics approach plays a crucial role in introducing metabolic pathways related to stage-specific of *Leishmania* parasite.

## Data Availability

Data of this study are included in the article and the primary data can be provided from the corresponding author.

## Abbreviations

SWATH-MS: Sequential window acquisition of all theoretical fragment ion spectra mass spectrometry
*L*: *Leishmania*
ITS1: Intrnal transcribed-spacer-1
NNN: Novy-nicolle-mc neal
MCODE: Molecular complex detection
COG: Cluster of orthologs groups
GO: Gene ontology
PPI: Protein–protein interaction
PCR–RFLP: Polymerase chain reaction-restriction fragment length polymorphism
FC: Fold change
2DE: Two dimensional electrophoresis
PTM: Post translational modification
FCS: Fetal calf serum
KEGG: Kyoto encyclopedia of genes and genomes
TCA: Tricarboxylic acid
KMP-11: Kinetoplastid membrane protein-11
